# REDD1 expression in podocytes facilitates renal inflammation and pyroptosis in streptozotocin-induced diabetic nephropathy

**DOI:** 10.1038/s41419-025-07396-4

**Published:** 2025-02-07

**Authors:** Siddharth Sunilkumar, Sandeep M. Subrahmanian, Esma I. Yerlikaya, Allyson L. Toro, Edward W. Harhaj, Scot R. Kimball, Michael D. Dennis

**Affiliations:** 1 Department of Cellular and Molecular Physiology, Penn State College of Medicine, Hershey, PA USA; 2 Department of Microbiology and Immunology, Penn State College of Medicine, Hershey, PA USA

**Keywords:** Diabetic nephropathy, Inflammasome, Diabetes complications

## Abstract

Sterile inflammation resulting in an altered immune response is a key determinant of renal injury in diabetic nephropathy (DN). In this investigation, we evaluated the hypothesis that hyperglycemic conditions augment the pro-inflammatory immune response in the kidney by promoting podocyte-specific expression of the stress response protein regulated in development and DNA damage response 1 (REDD1). In support of the hypothesis, streptozotocin (STZ)-induced diabetes increased REDD1 protein abundance in the kidney concomitant with renal immune cell infiltration. In diabetic mice, administration of the SGLT2 inhibitor dapagliflozin was followed by reductions in blood glucose concentration, renal REDD1 protein abundance, and immune cell infiltration. In contrast with diabetic REDD1^+/+^ mice, diabetic REDD1^−/−^ mice did not exhibit albuminuria, increased pro-inflammatory factors, or renal macrophage infiltration. In cultured human podocytes, exposure to hyperglycemic conditions promoted REDD1-dependent activation of NF-κB signaling. REDD1 deletion in podocytes attenuated both the increase in chemokine expression and macrophage chemotaxis under hyperglycemic conditions. Notably, podocyte-specific REDD1 deletion prevented the pro-inflammatory immune cell infiltration in the kidneys of diabetic mice. Furthermore, exposure of podocytes to hyperglycemic conditions promoted REDD1-dependent pyroptotic cell death, evidenced by an NLRP3-mediated increase in caspase-1 activity and LDH release. REDD1 expression in podocytes was also required for an increase in pyroptosis markers in the glomeruli of diabetic mice. The data support that podocyte-specific REDD1 is necessary for chronic NF-κB activation in the context of diabetes and raises the prospect that therapies targeting podocyte-specific REDD1 may be helpful in DN.

## Introduction

Diabetic nephropathy (DN) is one of the major complications in people with type 1 or type 2 diabetes and the most common cause of end-stage kidney disease [1]. As per the 2022 United States Renal Data System (USRDS) annual report, the prevalence of diabetes has increased to 35.6% in patients with chronic kidney complications [2]. Despite the improved prognosis of diabetes with the advent of sodium glucose cotransporter 2 (SGLT2) inhibitors and glucagon-like peptide-1 receptor agonists, a great number of patients with diabetes still develop renal failure and the risk of death remains high [3]; so much so that diabetes and chronic kidney disease are among the top 10 causes of mortality in the US [4]. This is in part due to a lack of understanding of the specific molecular events that contribute to diabetes-induced renal pathology.

Although the etiology of DN is complicated and multifactorial, it is widely accepted that inflammation plays a critical role in renal dysfunction. DN is often viewed as a chronic inflammatory disease and the role of innate and adaptive immune responses are recognized as important etiological components of DN pathogenesis [5]. The transcription factor nuclear factor κ-light-chain enhancer of activated B cells (NF-κB) plays a major role in mediating inflammation and immune function, with enhanced activity seen in the kidneys of diabetic patients and in preclinical models of diabetes [6, 7]. A complex network of extracellular perturbagens and signaling pathways are regulated by the NF-κB family of pleiotropic transcription factors [RelA (p65), RelB, c-Rel, p50, and p52] that act to promote the expression of a variety of pro-inflammatory cytokines, such as TNFα, IL-6, and IL-1β, as well as chemokines like CCL2 and RANTES. Inflammatory cytokines not only regulate immune responses, but are also cardinal effectors of renal injury. Increased circulating and local renal expression of pro-inflammatory cytokines and chemokines have been reported in diabetic patients and are associated with albuminuria and clinical markers of renal injury [8, 9].

Sterile inflammation in DN is often attributed to activation of the NOD-like receptor (NLR) family pyrin domain containing 3 (NLRP3) inflammasome complex in response to metabolic stimuli associated with diabetes [10, 11]. The NLRP3 inflammasome is unique among inflammasome complexes because it is sensitive to a wide range of stimuli. When activated, the NLRP3 inflammasome complex acts to not only process the maturation of interleukin (IL)-1β that contributes to an inflammatory response, but also causes pyroptosis, a pro-inflammatory form of lytic cell death [12]. Activation of the NLRP3 inflammasome has been closely linked with diabetic kidney disease in humans and in preclinical experimental models [10, 11, 13, 14]. NLRP3 inflammasome activation has been reported in a variety of renal cells including podocytes [14]. In fact, recent evidence supports immune cell-like functions of podocytes, as they produce chemokines that recruit immune cells and cytokines that drive their differentiation [11, 14, 15]. Shazad et al. demonstrated that suppression of NLRP3 specifically in podocytes is sufficient to attenuate renal injury and dysfunction in diabetic mice [11]. However, the specific signaling events that promote NLRP3 inflammasome activation and consequently the canonical and non-canonical signaling that is triggered in the context of DN remain to be fully defined.

The stress response protein REDD1 (Regulated in Development and DNA Damage 1; also known as DDIT4 or RTP801) is upregulated in the kidneys of diabetic patients, as well as in preclinical rodent models of diabetes [16, 17]. Our laboratory recently demonstrated that whole-body REDD1 deletion is sufficient to prevent albuminuria, renal injury, and podocyte loss in diabetic mice [16]. The significance of this observation is supported by a strong positive correlation between clinical indicators of renal disease and kidney REDD1 protein content in diabetic patients [17]. However, the mechanism responsible for reno-protection in diabetic REDD1 knockout mice remains to be fully established. Notably, REDD1 is required for the development of diabetes-associated inflammation [18]. REDD1 has been shown to promote and sustain NF-κB activation, thereby upregulating pro-inflammatory and immune responses in the context of disease [19–24]. To date, a role for REDD1 in diabetes-induced immune responses in the kidney has not been fully elucidated. Studies herein investigated the role of REDD1-dependent signaling in the development of renal inflammation.

## Material and methods

### Animal experiments

All procedures adhered to the National Institutes of Health Guide for the Care and Use of Laboratory Animals and ARRIVE guidelines, and were approved by the Penn State College of Medicine Institutional Animal Care and Use Committee. Mice were maintained as littermate cages (4 mice/cage) on a 12:12 h reverse light dark cycle with ad libitum access to food and water. At 6 weeks of age, littermate cages were randomly divided into treatment groups. Diabetes was induced in male mice by administering 50 mg/kg streptozotocin (STZ; Sigma Aldrich, St. Louis, MO, US) intraperitoneally (ip) over 5 consecutive days, and phenotype was confirmed by fasting blood glucose concentrations >250 mg/dL. Non-diabetic mice received sodium citrate buffer (0.01 M, pH 6) as a vehicle (Veh) control. Hyperglycemia was controlled via daily administration of 1 mg/kg Dapagliflozin (DG in 0.1% DMSO, ip; Selleck Chemicals, Houston, TX, USA) for 2 weeks beginning 14 weeks post-diabetes induction [25]. Male B6;129 REDD1^+/+^ and REDD1^−/−^ mice [26] were made diabetic as described. Podocyte-specific REDD1 knockout (PodKO) mice were generated by crossing hemizygous B6.Cg-Tg(NPHS2-cre)295Lbh/J (Stock #008205; The Jackson Laboratory, Bar Harbor, ME, USA) with REDD1^fl/fl^ mice [27] and administered STZ or Veh as described above. At 16 weeks of diabetes, all treatment indicators were removed from cages by a technician and after 4 h of fasting, one mouse from each group was euthanized under isoflurane anesthesia in a random order. Urine was collected from the bladder, and kidneys were analyzed as described below. Urine albumin and creatinine levels were measured as previously described [16].

### Immunohistology

Renal sections (6 µm) were cut from 10% formalin-fixed, paraffin-embedded (FFPE) kidneys and processed for immunohistochemistry (IHC) or immunofluorescence (IF) staining as described previously [16]. Antibodies are listed in Table S1. For IHC, sections were incubated with ImmPRESS HRP-conjugated secondary antibody and detected using 3,3ʹ-diaminobenzidine (Vector Laboratories, Newark, CA, USA). Tissue sections were counterstained with hematoxylin, mounted and micrographs were captured using an AmScope T720Q compound microscope (AmScope, Irvine, CA, USA). For IF labeling, tissue sections were incubated with appropriate secondary antibodies (Table S1) and counterstained with 1.6 μmol/L Hoechst 33342 (Thermo Fisher Scientific, Waltham, MA, USA). Immunocytochemistry analysis was done as previously described [28]. Cells were fixed with 4% PFA, permeabilized in 0.1% triton X-100, blocked with 5% BSA and then incubated with appropriate antibodies (Table S1). Cell nuclei were counterstained with 1 μM DAPI (Invitrogen, Carlsbad, CA, USA). All slides were mounted with Fluoromount aqueous mounting media (Sigma-Aldrich) and imaged with a Leica SP8 confocal laser microscope (Leica, Deerfield, IL, USA) using frame-stack sequential scanning.

### Flow cytometry

For flow cytometric analysis, cell suspensions of kidney tissue were made using Liberase digestion as previously described [29]. Briefly, kidneys were washed in cold PBS, minced finely and then shaken for 30 min at 37 °C in serum free RPMI medium containing 100 µg/ml Liberase (Roche, Basel, Switzerland) enzyme. Digestion was stopped by adding RPMI medium containing 10% fetal bovine serum, centrifuged, and resuspended in PBS. Cell debris was separated using Debris Removal Solution (Miltenyi Biotec, Bergisch Gladbach, Germany). Erythrocytes were lysed using RBC Lysis Buffer (eBioscience, San Diego, CA, USA) and washed with PBS. Samples were resuspended in Cell Staining Buffer (Biolegend, San Diego, CA, USA) and incubated in Fc Block (BD Biosciences, Franklin Lakes, NJ, USA) for 15 min prior to labeling with antibodies (Table S1) for 45 min. After washing, fixed cells were analyzed by flow cytometry using a BD LSRFortessa (BD Biosciences) instrument in Penn State College of Medicine’s Flow Cytometry Core (RRID:SCR_021134).

### Cell culture

Conditionally immortalized human podocytes (CIHP-1) were cultured as previously described [16]. CIHP-1 cells were cultured in RPMI 1640 media at 33 °C in 5% CO_2_, then differentiated for 10 days at 37 °C in 5% CO_2_ before treatments. Human leukemia monocytic THP-1 cells (ATCC TIB-202) were differentiated into macrophages using 50 ng/mL phorbol 12-myristate-13-acetate (Cayman Chemicals, Ann Arbor, MI, USA) for 48 h. CRISPR/Cas9 genome editing was used to generate a stable CIHP-1 REDD1 knockout (REDD1 KO) cell line [16]. Plasmids including a pCMV5 vector (EV), HA-tagged pCMV-HA-REDD1, or pBabe-GFP-IκB alpha-mut (IκBα super repressor; Addgene plasmid # 15264) were transiently transfected using Jet PRIME (Polyplus transfection, New York, NY, USA). To model hyperglycemia, cells were exposed to culture medium containing 30 mM glucose (HG) versus 5 mM glucose supplemented with 25 mM mannitol as an osmotic control (OC). Lactate dehydrogenase (LDH) released into cell culture supernatant was quantified after 48 h HG by LDH Cytotoxicity Assay Kit (Cayman Chemicals) following manufacturer’s instructions. To quantify NF-κB activity, differentiated CIHP-1 cells were co-transfected with the Renilla luciferase (Promega, Madison, WI, USA) and NF-κB-TATA-luciferase [30] plasmids. After 24 h, cells were exposed to hyperglycemic conditions, and luciferase activity was measured.

### Protein analysis

Nuclear or total proteins were extracted from cells or renal cortical tissue. Western blot analysis was carried out as previously described [19] with the appropriate antibodies (Table S1). Uncropped western blots are presented in the supplemental information. IL-1β protein was determined in culture medium or kidney homogenates by ELISA (DuoSet ELISA, R&D systems, Minneapolis, MN, USA). CCL2 recombinant protein (R&D systems, Minneapolis, MN, USA) was subjected to western blotting and CCL2 protein in cell (10^5^ cells) and tissue lysates were quantified (Fig. S1B,D & E). Nuclear NF-κB activity was quantified in renal tissue using a NF-κB p65 DNA-binding ELISA (TransAM NF-κB p65; Active Motif, Carlsbad, CA, USA). The FAM-FLICA Caspase-1 Kit (Bio-Rad Laboratories, Hercules, CA, USA) was used to detect active caspase-1 in podocyte cultures. Slides were counterstained with Hoechst 33342 (Thermo fisher Scientific), mounted, and imaged using a Leica SP8 confocal microscope (Leica Microsystems).

### Chromatin Immunoprecipitation

NF-κB p65 binding to the promoter region of the *CCL2* gene was determined by performing chromatin-immunoprecipitation (ChIP) using a Simple ChIP Plus Enzymatic ChIP Kit (Cell Signaling, Danvers, MA, USA) and quantitative PCR (ChIP-qPCR). Chromatin was crosslinked with proteins from CIHP-1 podocytes and the protein-chromatin complex was disrupted by ultrasonication. The soluble chromatin was subjected to an overnight incubation at 4 °C with either anti-p65 antibody or IgG (negative control), and protein G magnetic beads (Cell Signaling) were used for IP. RT-PCR analysis of the recovered DNA was performed with CCL2 primers (Table S2) encompassing the region of the human CCL2 promoter ( − 209 to −9; RefSeq accession NM_002982) [31]. Fold enrichment adjusted to the IgG controls was used to tabulate the results [32].

### PCR analysis

Total RNA was extracted, reverse transcribed, and subjected to quantitative real-time PCR (QuantStudio 12 K Flex Real-Time PCR System, Thermo Fisher Scientific, RRID:SCR_021098) with primers listed in Table S2. Mean cycle threshold values were determined. Change in mRNA expression relative to GAPDH mRNA was calculated.

### Transwell migration assay

Migration of THP-1 cells across Transwell inserts was measured as described [33]. Differentiated CIHP-1 were seeded into the lower chamber of the Transwell system (Corning, Kennebunk, ME, USA) and exposed to hyperglycemic conditions or osmotic control. Activated THP-1 cells were transferred into the top chamber of the inserts and allowed to migrate for 24 h. Cells attached to the bottom surface were fixed, stained, and imaged using a Nikon Eclipse TS100 inverted microscope (Nikon Instruments, Melville, NY, USA). Ten fields of view were imaged per sample, the number of migrated cells was quantified using ImageJ software, and counts were manually verified.

### Statistical analysis

Based on A priori power analysis of urinary albumin: creatinine ratio (ACR) in diabetic vs non-diabetic wild type mice from prior works [16, 34], an N of 4 was determined to yield statistically significant results (Effect size d = 2, α = 5%). Data are expressed as mean ± SD. Statistical analysis of data with more than two groups were analyzed by one-way or two-way ANOVA, with Tukey’s test for multiple comparisons used for pairwise analysis. The relationships between urine albumin to creatinine ratio (ACR) and blood glucose levels were tested by Spearman’s correlation analysis. Significance was defined as p < 0.05 for all analyses. Sample size for each experimental group and exact p-values for significantly different groups are listed in Table S3. Model assumptions were checked using the Shapiro-Wilk normality test and by visual inspection of residual plots.

## Results

### Diabetes-induced hyperglycemia promoted renal REDD1 content and immune cell infiltration in the kidney

**Fig. 1:**
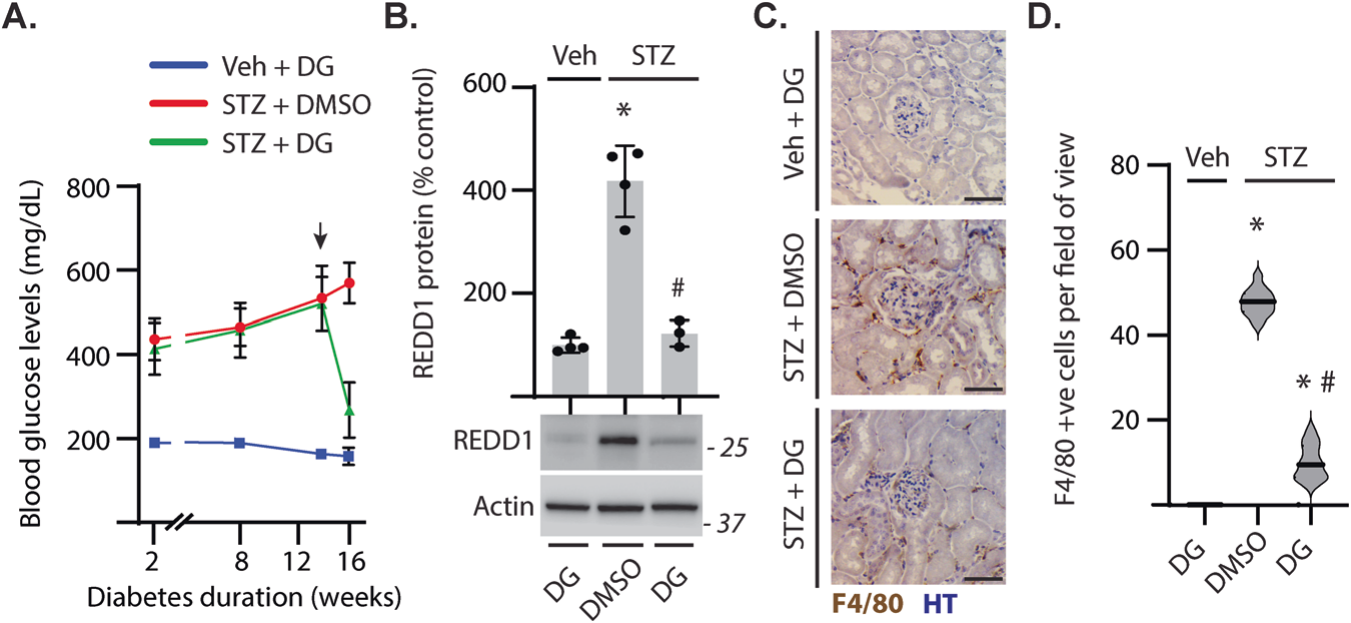
Hyperglycemia-induced REDD1 expression was associated with increased renal immune cell infiltration in diabetic mice. Diabetes was induced in mice by administration of streptozotocin (STZ). Analyses were performed 16 weeks after administration of STZ or vehicle (Veh). Hyperglycemia was controlled by daily administration of dapagliflozin (DG) beginning 14 weeks after diabetes induction. As a control for DG intervention, diabetic mice were administered a vehicle containing 0.1% DMSO. **A** Fasting blood glucose concentrations were measured. Arrow indicates the initiation of DG/DMSO intervention. **B** REDD1 and actin protein abundance was assessed in kidney cortical tissue homogenates by western blotting. Representative blots are shown. Protein molecular mass is indicated at *right* of each blot. Individual data points are plotted with values presented as means ± SD (*n* = 3–4). **C** F4/80 positive immune cells were identified in renal sections by immunohistochemistry (*brown*). Nuclei were counterstained with hematoxylin (HT, *blue*). Representative micrographs are shown (scale bar 50 µm). **D** F4/80 positive cells were quantified in 20 fields per section. Data distribution is represented by violin plot. Differences between groups were identified by one-way ANOVA. *p < 0.05 versus Veh; #*p* < 0.05 versus DMSO.

As compared to non-diabetic mice, fasting blood glucose levels were elevated in STZ-induced diabetic mice. Treatment with DG was followed by reduced hyperglycemia (Fig. 1A). STZ-diabetes increased REDD1 protein content in renal cortical homogenates (Fig. 1B) and enhanced renal immune cell infiltration (Fig. 1C, D). When diabetic mice were treated with DG, REDD1 protein content and immune cell infiltration in the kidneys were reduced (Fig. 1B–D). Together the data supports a role for diabetes-induced hyperglycemia in promoting renal REDD1 protein abundance and activation of the immune response in the kidneys.

### REDD1 ablation attenuated the diabetes-induced renal pro-inflammatory response

**Fig. 2:**
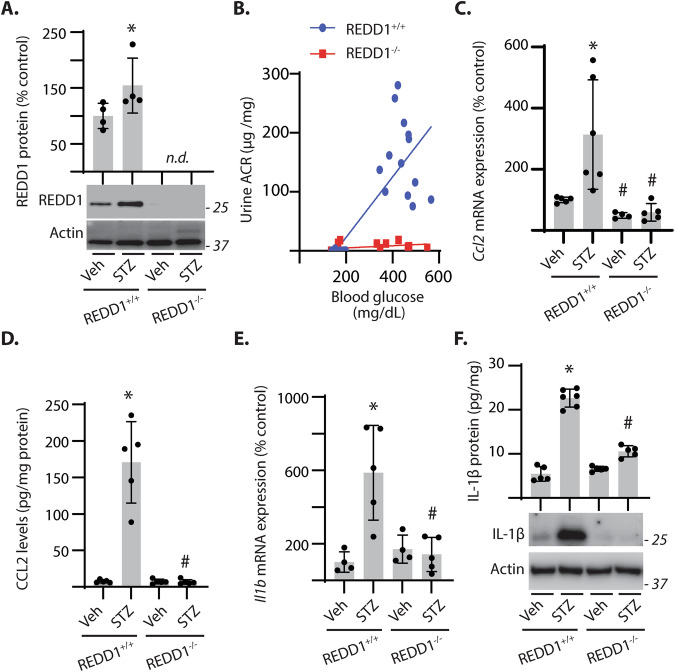
REDD1 was necessary for increased expression of inflammatory factors in the kidney of diabetic mice. Diabetes was induced in REDD1^+/+^ and REDD1^−/−^ mice by streptozotocin (STZ) administration. Non-diabetic control mice received vehicle (Veh). **A** REDD1 protein was evaluated in kidney cortical tissue homogenates by western blotting. Representative blots are shown. Molecular mass in kDa is indicated at *right* of each blot. **B** Correlation between fasting blood glucose and urine ACR is shown for REDD1^+/+^ mice (*blue*; Pearson *r* = 0.72; *p* < 0.0001) and REDD1^−/−^ mice (*red*; Pearson r = 0.55; *p* = 0.029). **C** *Ccl2* mRNA expression was quantified in kidney homogenates by qPCR. **D** CCL2 protein abundance was quantified in kidney homogenates by western blotting. **E** *Il1b* mRNA expression was quantified in kidney homogenates by qPCR. **F** IL-1β protein levels were determined in kidney homogenates by western blotting and quantified by ELISA. Individual data points are plotted with values presented as means ± SD (*n* = 4–6). Differences between groups were identified by two-way ANOVA. *p < 0.05 versus Veh; #*p* < 0.05 versus REDD1^+/+^. n.d., not detected.

To evaluate the role of REDD1 in renal inflammation, wild-type and REDD1-deficient mice were administered STZ. REDD1 protein in the kidneys of STZ-diabetic REDD1^+/+^ mice was increased compared to non-diabetic controls (Fig. 2A). Elevated blood glucose levels were observed in both genotypes with STZ administration; however, a positive correlation between blood glucose concentrations with urine ACR was only observed in REDD1^+/+^ but not REDD1^−/−^ mice (Fig. 2B**;** Pearson r: REDD1^+/+^ = 0.77 *vs* REDD1^−/−^ = 0.4**)** [16]. Global REDD1 deletion attenuated STZ-diabetes induced renal mRNA expression of proinflammatory genes including *Ccl5, Vegfa*, and *Icam-1* (Fig S1A). Monocyte chemoattractant protein-1 (MCP-1/CCL2) is a ligand of C-C motif chemokine receptor 2 (CCR-2) and a predictive biomarker for the development of DN [35]. Diabetes increased *Ccl2* mRNA expression (Fig. 2C) and protein levels (Fig. 2D, S1B) in the kidneys of REDD1^+/+^ mice. By contrast, CCL2 was not increased in the kidneys of diabetic REDD1^−/−^ mice. Similarly, diabetes also increased renal interleukin 1β mRNA (Fig. 2E) and protein (Fig. 2F) in a REDD1-dependent manner. The data support that REDD1 is necessary for enhanced renal inflammatory cytokine and chemokine expression in DN.

### REDD1 deletion attenuated renal immune cell infiltration in diabetic mice

**Fig. 3:**
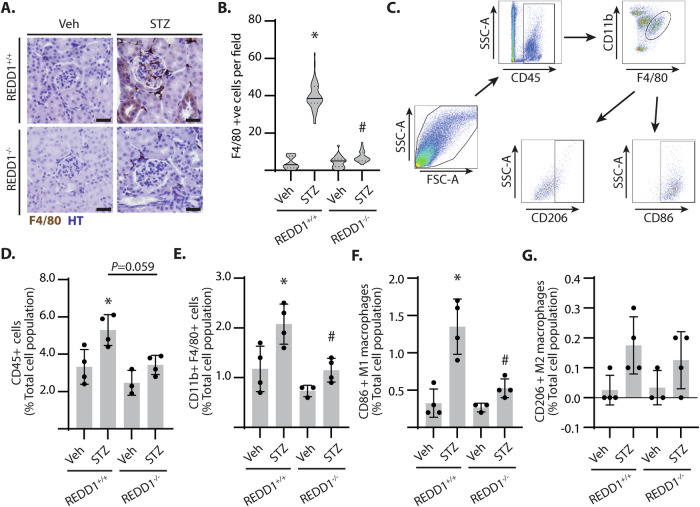
REDD1 was required for renal infiltration of pro-inflammatory macrophages in diabetic mice. **A** Kidney sections from diabetic (STZ) and non-diabetic (Veh) mice were immunolabeled for F4/80 (*brown*) and counterstained with hematoxylin (HT, *blue*). Representative micrographs are shown (scale bar 50 µm). **B** F4/80 positive cells in *A* were quantified in 20 fields per renal section. Data distribution is represented by violin plot. **C**–**G** Flow cytometry was used to identify renal immune cell populations. Gating strategy to determine immune cell populations in the kidney is shown (**C**). Populations of CD45+ cells (**D**), CD11b + F4/80+ macrophages (**E**), CD86 + M1 macrophages (**F**) and CD206 + M2 macrophages (**G**) were determined. Individual data points are plotted with values presented as means ± SD (*n* = 3–4). Differences between groups were identified by two-way ANOVA. *p < 0.05 versus Veh; #*p* < 0.05 versus REDD1^+/+^.

Due to the critical role of macrophage infiltration in DN pathogenesis [36], renal immune cell infiltrates were evaluated by labeling for F4/80 (Fig. 3A). The number of F4/80+ cells in the renal cortex of diabetic REDD1^+/+^ mice was increased as compared to non-diabetic controls (Fig. 3B). Diabetes-induced immune cell infiltration was absent in REDD1^−/−^ mice. Infiltrating immune cells were characterized by flow cytometry (Fig. 3C, S2). STZ-diabetes increased infiltrating CD45+ cells within the kidney (Fig. 3D), and REDD1 deletion prevented this effect. Within the population of CD45+ cells, an increase in CD11b + F4/80+ macrophages was observed in the kidneys of diabetic REDD1^+/+^ mice, but not in diabetic REDD1^−/−^ mice (Fig. 3E). Macrophages were further characterized based on polarization as CD86+ (M1) or CD206+ (M2) cells. REDD1 ablation prevented the diabetes-induced increase in CD86 + M1 macrophages (Fig. 3F). No significant changes were observed in CD206+ macrophage populations (Fig. 3G). These data support that REDD1 expression is necessary for elevated pro-inflammatory innate immune responses in the kidneys of diabetic mice.

### REDD1 expression was required for NF-κB activation in podocytes

**Fig. 4:**
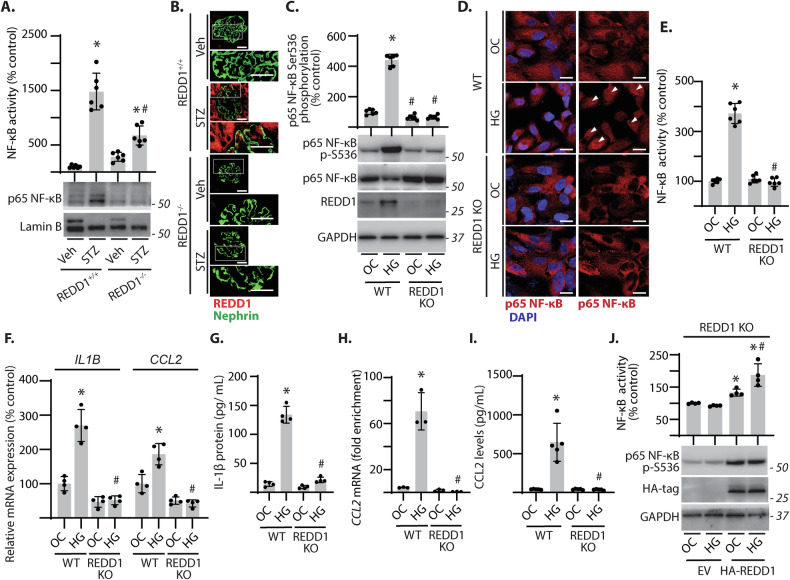
REDD1 deletion prevented diabetes-induced NF-κB activation in the kidney. **A**, **B** Diabetes was induced in REDD1^+/+^ and REDD1^−/−^ mice by administration of streptozotocin (STZ). Non-diabetic control mice received vehicle (Veh). **A** Nuclear isolates were prepared from kidney homogenates. NF-κB and Lamin B were examined in nuclear isolates by western blotting and NF-κB activity was quantified by DNA-binding ELISA. Representative blots are shown with protein molecular mass in kDa indicated at *right* of each blot. **B** REDD1 (*red*) and Nephrin (*green*) were visualized in kidneys by immunofluorescence microscopy. White box indicates area shown at increased magnification. Representative micrographs are shown (scale bar 50 μm). **C**–**I** Wild-type (WT) and REDD1 knockout (KO) CIHP-1 were exposed to culture media containing either 30 mM glucose (HG) or 5 mM glucose plus 25 mM mannitol (OC) for 48 h. NF-κB phosphorylation at S536 and REDD1 protein abundance was determined in cell lysates by western blotting (**C**). Nuclear localization of NF-κB p65 (*white arrowheads*) was evaluated by immunofluorescence (**D**). Nuclei were visualized with DAPI (scale bar 25 μm). NF-κB activity was measured in lysates from cells expressing NF-κB firefly luciferase/*Renilla* luciferase reporter plasmids by dual luciferase assay (**E**). Relative expression of *IL1B* and *CCL2* mRNA were determined by qPCR (**F**). IL-1β secreted into culture media was determined by ELISA (**G**). Chromatin immunoprecipitation (ChIP)-PCR analysis was carried out in WT and REDD1 KO podocytes to determine binding of p65 NF-κB to the promoter region of the CCL2 gene (**H**). CCL2 protein levels were determined in cell lysates by western blotting (**I**). **J** NF-κB p65 phosphorylation and NF-κB luciferase reporter activity was evaluated in REDD1 KO cells expressing either an empty vector control (EV) or hemagglutinin (HA)-tagged REDD1. Individual data points are presented as means ± SD (*n* = 4–6). Differences between groups were identified by two-way ANOVA. *p < 0.05 versus Veh or NG; #*p* < 0.05 versus REDD1^+/+^, WT, or EV.

NF-κB signaling involves the degradation of the inhibitor of κB (IκB) to facilitate the phosphorylation and nuclear translocation of NF-κB. Degradation of IκBα (Fig. S3A) and enhanced nuclear localization of NF-κB and increased NF-κB activity were observed in the kidneys of diabetic REDD1^+/+^ mice (Fig. 4A). As compared to REDD1^+/+^ mice, diabetes-induced NF-κB activation was reduced in the kidneys of REDD1^−/−^ mice. Prior reports suggest a role for podocytes in mediating inflammatory responses in diabetes [11, 37]. REDD1 partially colocalized with the podocyte marker nephrin in the kidneys of diabetic mice (Fig. 4B). To investigate the role of REDD1 in podocytes, CIHP-1 cells were exposed to hyperglycemic culture conditions. Hyperglycemic conditions promoted both the degradation of IκBα **(**Fig. S3B**)** and phosphorylation of the p65 NF-κB subunit at S536 (Fig. 4C), NF-κB nuclear localization (Fig. 4D), and NF-κB luciferase reporter activity (Fig. 4E) in podocytes. CRISPR-Cas9-mediated REDD1 deletion prevented these effects in CIHP-1 cells (Fig. 4C–E). To assess specificity of NF-κB signaling in podocytes, CIHP cells expressing either EV or an IκBα mutant plasmid were exposed to hyperglycemic conditions. High glucose-induced expression of mRNAs encoding the NF-κB target genes *IL1B* and *CCL2* was attenuated with suppressed activation of NF-κB (Fig. S3C). Notably, expression of these genes were upregulated in wild-type cells, but not in REDD1-deficient cells, upon exposure to hyperglycemic conditions (Fig. 4F). REDD1 deletion also attenuated mRNA expression of pro-inflammatory genes including *TNFA*, *VEGFA*, and *ICAM-1* in podocytes exposed to hyperglycemic conditions **(**Fig. S1C**)**. Hyperglycemic conditions also upregulated IL-1β protein released into media in a manner that was dependent on REDD1 (Fig. 4G). ChIP PCR analysis indicated increased binding of p65 to the *CCL2* promoter in wild-type cells exposed to hyperglycemic conditions, but not in REDD1-deficient cells exposed to hyperglycemic conditions (Fig. 4H). We also observed a REDD1-dependent increase in CCL2 protein in cell lysates upon exposure to hyperglycemic conditions (Fig. 4I, S1D). To confirm the role of REDD1 in NF-κB activation in podocytes, REDD1 was rescued in REDD1 knockout podocytes by expression of an HA-tagged REDD1. HA-REDD1 enhanced NF-κB p65 phosphorylation at S536 in REDD1 knockout podocytes and restored the increase in NF-κB activity upon exposure to hyperglycemic conditions (Fig. 4J). These data support that REDD1 is both necessary and sufficient to increase NF-κB activation in podocytes under hyperglycemic conditions.

### Podocyte-specific deletion of REDD1 protected against macrophage infiltration in DN

**Fig. 5:**
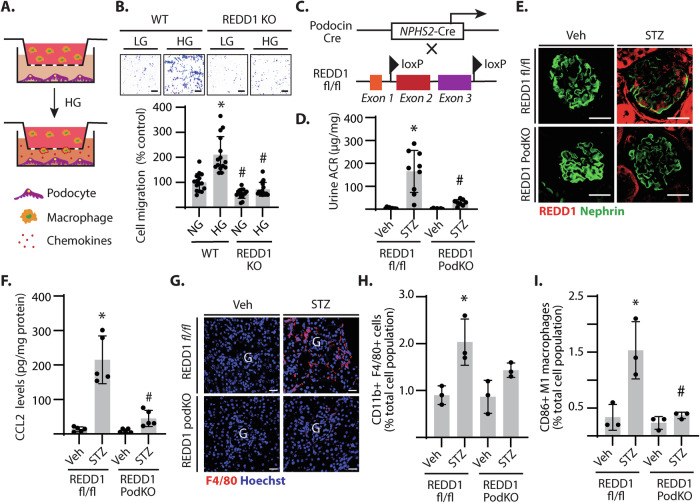
Podocyte-specific expression of REDD1 was required for increased renal macrophage infiltration in the kidney of diabetic mice. **A**, **B** Differentiated wild-type (WT) and REDD1 knockout (KO) CIHP-1 were exposed to culture media containing either 30 mM glucose (HG) or 5 mM glucose plus 25 mM mannitol as an osmotic control (OC) for 48 h. Transwell migration assay was used to evaluate chemotaxis in a co-culture model with CIHP-1 and THP-1 macrophages (**A**). Macrophages were stained with crystal violet and cells that migrated across the Transwell were counted (**B**). **C** Cre-lox recombination was used to achieve conditional podocyte-specific REDD1 knockout (REDD1 PodKO). **D**–**H** Diabetes was induced in REDD1^fl/fl^ and REDD1 PodKO mice by streptozotocin (STZ) administration. Non-diabetic groups were administered a vehicle (Veh) control. All assessments were performed after 16 weeks of diabetes. Urine albumin to creatinine ratio (ACR) was determined (D). Kidney sections from diabetic and non-diabetic mice were immunolabeled for REDD1 (*red*) and the podocyte marker Nephrin (*green*) (**E**). Protein abundance of CCL2 was determined in renal homogenates by western blotting (**F**). Representative blots are shown with protein molecular mass in kDa indicated at *right* of each blot. Kidney sections were immunolabelled for F4/80 (*red*) and nuclei were counterstained with Hoechst 33342 (*blue*) (**G**). Representative micrographs (scale bar 50 µm) are shown. Immune cell populations of CD11b + F4/80+ macrophages (**H**) and CD86 + M1 macrophages (**I**) were determined by flow cytometry. Individual data points are plotted. Significance was analyzed by two-way ANOVA and pairwise comparisons were made using the Tukey’s test for multiple comparisons. *p < 0.05 versus OC or Veh; #, p < 0.05 versus WT or REDD1^fl/fl^.

To evaluate the role of podocyte-specific REDD1 in macrophage infiltration, wild-type and REDD1-deficient CIHP-1 cells were exposed to hyperglycemic conditions. A Transwell migration assay was then used to assess macrophage chemotaxis by podocytes (Fig. 5A). Increased transmigration of THP-1 macrophages was observed when co-cultured with wild-type CIHP-1 cells exposed to hyperglycemic conditions (Fig. 5B). However, a similar increase in chemotaxis was not observed when macrophages were co-cultured with REDD1-deficient CIHP-1 cells exposed to hyperglycemic conditions. Thus, REDD1 deletion in podocytes attenuated both the increase in chemokine expression and macrophage chemotaxis under hyperglycemic conditions. To evaluate the role of podocyte-specific REDD1 expression in the kidney, targeted REDD1 deletion in podocytes was carried out by NPHS2-cre directed excision of exons 2 and 3 from the REDD1 gene (Fig. 5C) [34]. To evaluate a role for podocyte-specific REDD1 expression in diabetes-induced renal inflammatory responses, REDD1^fl/fl^ and REDD1 PodKO mice were administered STZ. After 16 weeks of STZ-induced diabetes, increased urine ACR was observed in REDD1 ^fl/fl^ mice but not in REDD1 PodKO mice (Fig. 5D). Urine ACR positively correlated with elevated fasting blood glucose concentrations in REDD1^fl/fl^ mice (Fig. S4A, Pearson r = 0.812). As compared to REDD1^fl/fl^ mice, the linear correlation between urine ACR and blood glucose was reduced in REDD1 PodKO mice (Pearson r = 0.646). REDD1 protein was increased throughout the kidneys of diabetic REDD1^fl/fl^ mice and colocalized with the podocyte marker nephrin within the glomerulus (Fig. 5E). REDD1 protein expression was absent within glomeruli and attenuated in tubules of diabetic REDD1 PodKO mouse kidneys, as compared to diabetic REDD1^fl/fl^ mice. Moreover, increased CCL2 protein levels (Fig. 5F, S1E) and F4/80+ immune cell infiltration (Fig. 5G) were observed in the kidneys of diabetic REDD1^fl/fl^ mice, but not diabetic REDD1 PodKO mice. Flow cytometry analysis (Fig. S4B) revealed a large proportion of the infiltrating CD45+ immune cells in diabetic REDD1^fl/fl^ mice to be macrophages (CD11b + F4/80 + ; Fig. 5H) that were further characterized as pro-inflammatory CD86 + M1 macrophages (Fig. 5I). As compared to the kidney of REDD1^fl/fl^ mice, fewer F4/80+ cells were observed in diabetic REDD1 PodKO mice and the M1 macrophage population was reduced. The data support that REDD1 expression specifically in podocytes promotes renal infiltration of pro-inflammatory macrophages in the context of diabetes.

### Podocyte-specific REDD1 deletion attenuated activation of the NLRP3 inflammasome and pyroptosis in DN

**Fig. 6:**
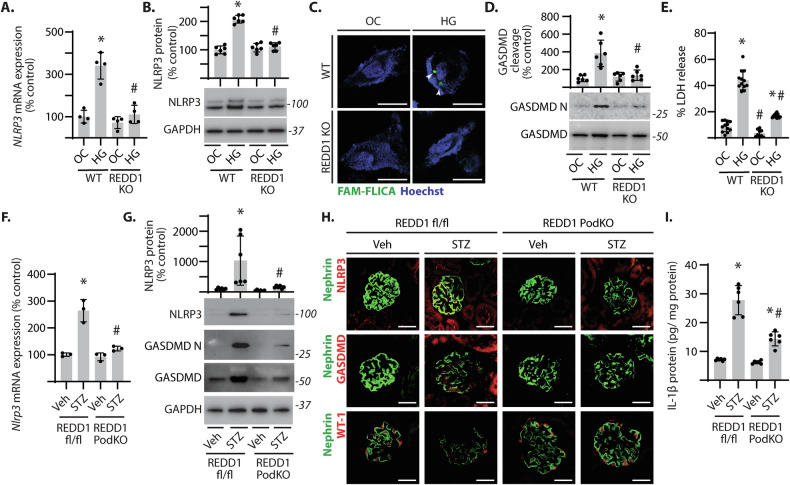
REDD1 expression in podocytes was required for NLRP3 inflammasome activation in diabetic mice. **A**–**E** Differentiated wild-type (WT) and REDD1 knockout (KO) CIHP-1 cells were exposed to culture media containing either 30 mM glucose (HG) or 5 mM glucose plus 25 mM mannitol as an osmotic control (OC) for 48 h. Relative expression of *NLRP3* mRNA was determined in cell lysates by qPCR (**A**). NLRP3 protein content relative to GAPDH was estimated by western blotting (**B**). Representative blots are shown with molecular mass in kDa indicated to the *right* of the blot. Active caspase-1 (*green*) was visualized by immunofluorescence microscopy using FAM-YVAD-FMK FLICA (fluorescently labeled inhibitor of caspase) probe (**C**; scale bar 25 µm). Gasdermin D (GSDMD) N-terminal cleavage (GSDMD-N) was determined in cell lysates by western blotting (**D**). LDH released into cell culture supernatant was quantified (**E**). *F-I*, Diabetes was induced in REDD1^fl/fl^ and REDD1 PodKO mice by streptozotocin (STZ) administration. Non-diabetic mice were administered a vehicle (Veh) control. *Nlrp3* mRNA expression in glomerular isolates was quantified by qPCR (F). NLRP3 and GSDMD protein in glomerular isolates were determined by western blotting (**G**). Immunofluorescence microscopy was used to determine colocalization of NLRP3, GSDMD, and WT-1 with the podocyte marker nephrin (**H**). IL-1β protein content in renal homogenates was quantified by ELISA (**I**). Representative micrographs (scale bar 50 µm) are shown. Individual data points are plotted. Significance was analyzed by two-way ANOVA and pairwise comparisons were made using the Tukey’s test for multiple comparisons. *p < 0.05 versus OC or Veh; #*p* < 0.05 versus WT or REDD1^fl/fl^.

Under metabolic stress, cells undergo pyroptosis, which is a pro-inflammatory form of cell death characterized by increased caspase-1 activity, Gasdermin D (GSDMD) cleavage, and lactate dehydrogenase (LDH) release [38]. In podocyte cultures, exposure to high glucose concentrations increased *NLRP3* mRNA expression (Fig. S3C), and suppression of NF-κB with the IκBα super repressor mutant plasmid attenuated this effect. To investigate the role of REDD1 in NLRP3 activation and pyroptosis, differentiated wild-type and REDD1-deficient podocytes were exposed to hyperglycemic conditions. *NLRP3* mRNA expression (Fig. 6A) and NLRP3 protein abundance (Fig. 6B) were increased in wild-type podocytes exposed to hyperglycemic conditions, but not in podocytes deficient for REDD1. In podocytes exposed to hyperglycemic conditions, there was increased caspase-1 activity (Fig. 6C), GSDMD cleavage (Fig. 6D), and LDH release (Fig. 6E) in a manner that was dependent on REDD1. To investigate if REDD1 expression in podocytes was necessary for diabetes-induced pyroptosis, kidneys and glomerular isolates from STZ-diabetic and non-diabetic REDD1^fl/fl^ and REDD1 PodKO mice were assessed. As compared to non-diabetic mice, *Nlrp3* mRNA expression (Fig. 6F) and NLRP3 protein content (Fig. 6G) were increased in glomeruli isolated from diabetic REDD1^fl/fl^ mice. We also observed an increase in GSDMD protein in glomeruli of diabetic REDD1^fl/fl^ mice compared to non-diabetic mice (Fig. 6G**)**. However, increases in NLRP3 and GSDMD were both blunted in glomeruli isolated from diabetic REDD1 PodKO mice. Immunofluorescence microscopy also showed an increased colocalization of NLRP3 and GSDMD with the podocyte marker nephrin in diabetic REDD1^fl/fl^ mice, but not in diabetic REDD1 PodKO mice (Fig. 6H). In support of a role for podocyte-specific REDD1 expression in podocyte loss with STZ-diabetes, the reduction in nephrin and WT-1 staining in diabetic REDD1^fl/fl^ mice was reduced in diabetic REDD1 PodKO mice (Fig. 6H). We also observed that podocyte-specific REDD1 deletion attenuated the increase in IL-1β levels in kidney homogenates from diabetic mice (Fig. 6I). Together, the data are consistent with a role for diabetes-induced REDD1 expression in mediating NLRP3-associated pyroptotic cell death in podocytes.

## Discussion

**Fig. 7:**
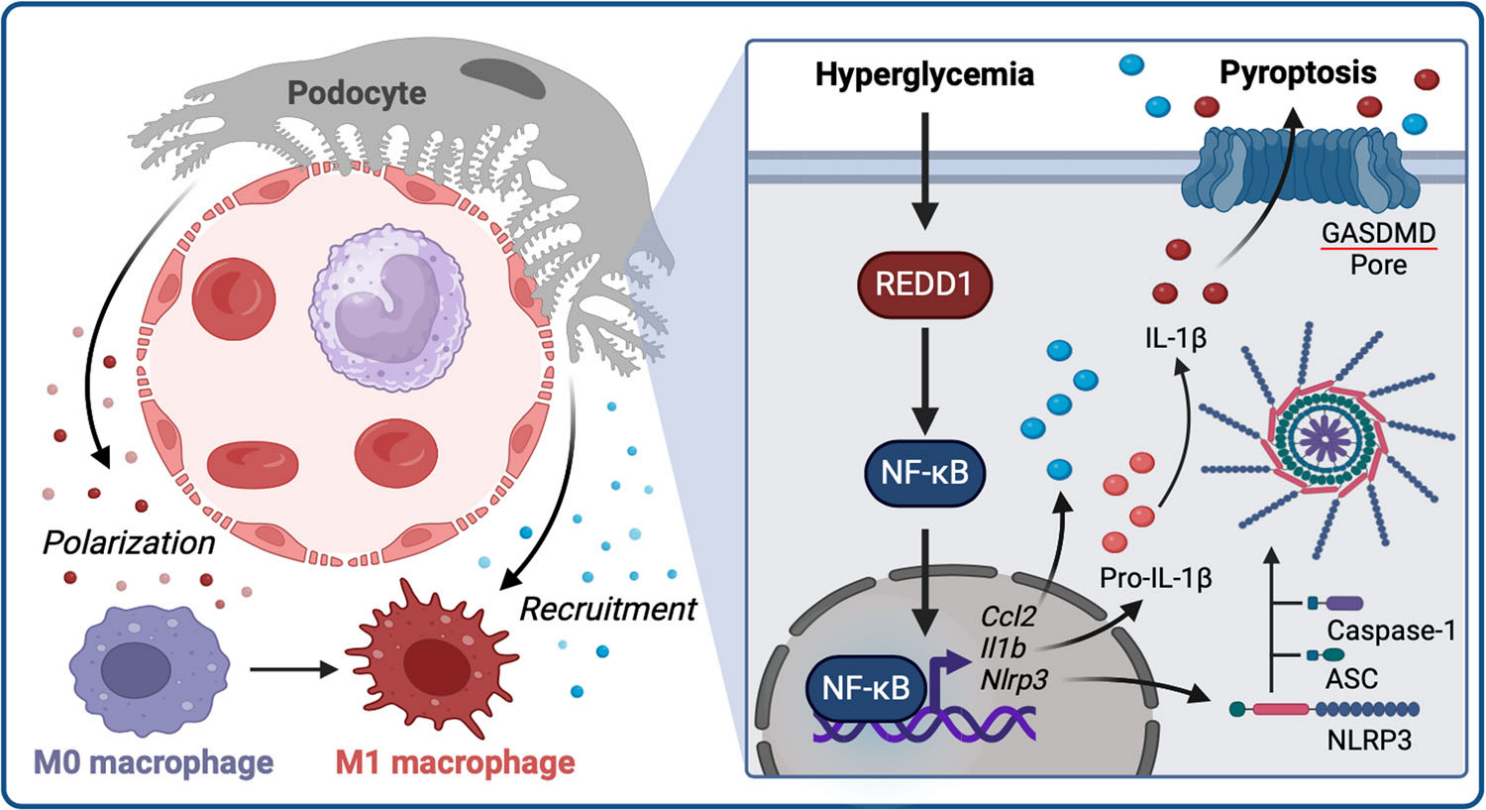
Working model for the role of podocyte-specific REDD1 expression in facilitating renal inflammation and pyroptosis in diabetic nephropathy. Diabetes-induced hyperglycemia enhances REDD1-dependent activation of NF-κB signaling in podocytes, resulting in increased cytokine and chemokine production. Podocyte-specific expression of REDD1 is required for kidney immune cell infiltration, macrophage polarization, and NLRP3 inflammasome-associated pyroptosis in diabetes.

Studies from the last two decades support a critical role for inflammation in the etiology of DN [39]. Herein, we investigated the role of REDD1 in diabetes-induced renal inflammation. Diabetes increased renal NF-κB activation and enhanced pro-inflammatory cytokine expression, with augmented renal infiltration of M1 pro-inflammatory macrophages in a manner that was dependent on REDD1. REDD1 was necessary and sufficient to promote NF-κB activation in podocytes exposed to hyperglycemic conditions. Importantly, the deletion of REDD1 specifically in podocytes attenuated macrophage infiltration in the kidneys of diabetic mice. Overall, the studies support a model wherein REDD1 expression in podocytes promotes NF-κB- and NLRP3-mediated inflammatory responses in the kidney including podocyte pyroptosis and the recruitment and polarization of macrophages in DN (Fig. 7).

Hyperglycemia is a determining factor in the development and progression of DN [40, 41]. Herein, hypo-insulinemia was induced in mice by administration of STZ, resulting in secondary hyperglycemia. While STZ is a valuable tool for modeling diabetic complications in genetically manipulated mice, it is important to note that strain-dependent variability in renal responses to STZ have been reported. With STZ administration, the C57BL/6 and B6;129 strains used in this study develop mild pathological changes in the kidney with mild-to-moderate albuminuria, as compared to other inbred mouse lines (e.g., DBA/2, KK-H1J) [42–44]. Importantly, the STZ-diabetic C57BL/6 mouse strain continues to be a helpful experimental paradigm to investigate diabetes-associated renal inflammation [45–47]. REDD1 levels are elevated in the kidneys of diabetic patients and in preclinical murine models of type 1 and type 2 diabetes [16, 17]. Upregulation of REDD1 occurs in multiple cell types exposed to diabetogenic conditions [16, 17, 27, 48, 49]. Normalization of blood glucose concentrations by SGLT2 inhibition was followed by attenuated REDD1 protein abundance in the kidney of diabetic mice concomitant with a reduction in immune cell infiltration. The observation builds on prior studies demonstrating increased REDD1 in renal cell cultures exposed to hyperglycemic conditions [16, 17, 49].

A growing body of research demonstrates that REDD1 controls critical cellular and metabolic functions [50], and is vital in the pathogenesis of metabolic disorders including diabetic retinopathy [19, 20, 27, 48] and nephropathy [16, 17, 49]. In the past decade, increasing evidence supports a pro-inflammatory role for REDD1 [19, 20, 22, 23, 49]. Chronic low-grade inflammation and activation of the innate immune response are integral to the pathogenesis of diabetes and its complications [51]. Inflammatory mediators like IL-1β, IL6 and CCL2 are upregulated in the kidneys of diabetic patients and act as pathogenic mediators in DN [9, 52]. Our data agree with these works and advance the understanding of mechanisms whereby REDD1 drives immune signaling in the context of DN. Specifically, REDD1 was necessary for activation of NF-κB, increased expression of cytokines and chemokines, and immune cell infiltration in the kidneys of diabetic mice. Lee et al. previously reported that REDD1 plays a role in the recruitment of immune cells into adipose tissue in murine model an obesity [22]. The data here support that REDD1 has a similar role in the recruitment of M1 pro-inflammatory macrophages into the kidney in the context of diabetes.

Podocyte dysfunction and loss is an early event in DN pathogenesis and predicts diabetic kidney injury [53]. Damaged podocytes produce inflammatory cytokines and chemokines that drive immune cell recruitment and glomerular inflammation [11, 49]. Activation of inflammatory pathways in non-hematopoietic kidney resident cells including podocytes promote inflammatory processes that aggravate renal injury in DN [14]. Indeed, podocyte-specific suppression of the NLRP3 inflammasome prevents diabetes-induced proteinuria [11]. Herein, REDD1 was required for NF-κB activation and the production of inflammatory cytokines and chemokines by podocytes. This advances findings from Wang et al. showing that REDD1 knockdown attenuates expression of TNFα, IL6, and IL-1β in podocyte cultures exposed to hyperglycemic conditions [49]. Additionally, in vitro transmigration assays demonstrated that hyperglycemia-induced REDD1 in podocytes was required for macrophage chemotaxis to the site of inflammatory injury. Importantly, in mice with podocyte-specific REDD1 deletion, diabetes failed to increase immune cell infiltration and renal recruitment of M1 pro-inflammatory macrophages. Notably, the attenuated inflammatory response within glomeruli observed with podocyte-specific REDD1 deletion correlated with preserved glomerular architecture and filtration function, as well as reduced podocyte loss [34].

Independent investigations have demonstrated that canonical and non-canonical activation of the inflammasome is a characteristic event in diabetic complications [38]. In the context of diabetic kidney disease, studies have shown that excessive cell pyroptosis mediated by caspase-1-associated canonical [54] cleavage of GSDMD (as well as caspase-11/4 non-canonical GSDMD cleavage [10]) promotes podocyte damage and renal immune cell infiltration. Moreover, in preclinical models of DN, podocyte-specific activation of the NLRP3 inflammasome is both necessary and sufficient to promote glomerular dysfunction and kidney damage [11]. In recent years, investigations delineating the regulation of NLRP3 inflammasome activation have implicated a role for REDD1 in both priming and activation of the inflammasome complex [21, 23, 55]. Prior work from our laboratory demonstrated a role for REDD1 in NF-κB-dependent NLRP3 inflammasome activity in the context of diabetic retinopathy [21]. The findings presented herein extend these prior studies and demonstrate that REDD1 expression in podocytes is required for NF-κB-dependent NLRP3 inflammasome activation and subsequent induction of pyroptosis in experimental models of diabetes.

A major limitation of the current standards of care for DN is that they predominantly focus on controlling blood glucose levels and fail to address the specific underlying cause of DN. The studies here delineate specific molecular events that contribute to renal inflammation caused by diabetes. Podocytes perform immune-surveillance functions and initiate immune responses that make the glomerular filtration barrier vulnerable to inflammatory disorders like DN [11]. The proof-of-concept studies here are consistent with a mechanism of action whereby REDD1 drives renal injury by promoting NF-κB activation in podocytes, thereby enhancing the renal pro-inflammatory immune response to diabetes. Given that REDD1 expression is also upregulated in acute kidney injury (AKI) [56, 57], interventions targeting REDD1 in the context of nephropathies including AKI and DN could improve current treatment paradigms. Novel podocyte-centric therapies like PS-001 [58], which recently entered clinical development, also offer great promise in developing therapeutics that can suppress REDD1 specifically in podocytes to combat proteinuric kidney disease.

## Supplementary information

Supplemental Materials

## Data Availability

All primary data including original western blots and immunofluorescent images supporting the findings of this study are presented within the manuscript or in the supplemental information.
