# Supplementary material for: REDD1 expression in podocytes facilitates renal inflammation and pyroptosis in streptozotocin-induced diabetic nephropathy
Source: Cell Death Dis. 2025 Feb 7;16(1):79. doi: 10.1038/s41419-025-07396-4 (PMC11806006; doi:10.1038/s41419-025-07396-4)

Supplemental Information

Stress response protein REDD1 expression in podocytes facilitates renal inflammation and pyroptosis in streptozotocin-induced diabetic nephropathy

Sunilkumar et al.

Table of Contents:

1. Table S1: Antibody information
2. Table S2: PCR oligonucleotide sequence
3. Table S3: Descriptive statistical analysis
4. Figure S1. Effect of REDD1 on proinflammatory cytokine expression
5. Figure S2. Flow cytometry analysis of REDD1$^{+/+}$ vs. REDD1$^{-/-}$
6. Figure S3. Effect of REDD1 on NF-κB signaling
7. Figure S4. Blood glucose: ACR correlation and Flow cytometry analysis of REDD1 fl/fl vs. REDD1 PodKO
8. Original western blots
9. Immunofluorescence individual channels

Table S1. Antibody list

Antibody list					
Assay	Antibody	Source	Catalog #	Lot#	Dilution
Western blotting	REDD1	ProteinTech	10638-1-AP	95508	1:500
	NLRP3	Invitrogen	PA5-79740	ZE4324561	1:1000
	phospho p65 NF-κB (Ser 536)	Cell Signaling	3033	17	1:1000
	p65 NF-κB		8242	8	1:1000
	CCL2/MCP-1		2029	2	1:1000
	IL-1β		12242	4	1:1000
	Gasdermin D		39754		1:1000
	Cleaved Gasdermin D (Asp275)		36425	4	1:1000
	HA-tag		3724	10	1:1000
	Actin		4970	19	1:1000
	Lamin B	Santa Cruz	sc6216	C1307	1:1000
	GAPDH		sc-47724	H1021	1:2000
	Goat anti-Rabbit IgG h+l-HRP	Bethyl laboratories	A120-101	44	1:10000
	Goat anti-mouse IgG h+l-HRP		A90-116	43	1:10000
	Rabbit anti-goat IgG h+l-HRP		A50-100P	29	1:10000
IF / IHC	REDD1	ProteinTech	10638-1-AP	110532	1:250
	F4/80	Cell Signaling	30325	3	1:200
	NF-κB		8242	8	1:500
	Cleaved Gasdermin D (Asp275)		36425	4	1:500
	NLRP3		PA5-79740	ZE4324561	1:500
	Nephrin	R&D Biotech	AF3159	CBIK0422122	1:500
	Donkey anti-Rabbit Alexa Fluor 647	Jackson laboratories	711-605-152	164324	1:500
	Donkey anti-goat Alexafluor 488		705-546-147	122088	1:500
Flow Cytometry	APC-Cy 7 Rat anti-Mouse CD45	BD Biosciences	561037	3041708	1:100
	PerCP-Cy 5.5 Rat anti-Mouse CD11b		561114	3079694	1:100
	Alexa Fluor 647 Rat anti-Mouse F4/80		565854	3115393	1:100
	PE Rat anti-Mouse CD86		561963	3145661	1:100
	Alexa Fluor 488 Rat anti-Mouse CD206		568807	2279205	1:100

Table S2. PCR oligonucleotide sequence

PCR primer list			
Species	Target	Forward sequence (5' - 3')	Reverse sequence (5' - 3')
Mouse	*Ccl2*	CACTCACCTGCTGCTACTCA	GCTTGGTGACAAAAACTACAGC
	Il1b	CACTACAGGCTCCGAGATCAAC	CCCCTGGAGATTGAGCTGTCTGC
	Nlrp3	CTCCAACCATTCTCTGACCAG	ACA GATT GAA GTA AGG CCG G
	Ccl5	TGCTGCTTTGCCTACCTCTC	TCCTTCGAGTGACAAACACGA
	Vegfa	GAGAGAGGCCGAAGTCCTTT	TTGGAACCGGCATCTTTATC
	Icam1	AGCCTCCGGACTTCGATCT	TGTTTGTGCTCTCCTGGGTC
	Gapdh	GGTGGTCTCCTCTGACTTCAACA	GTTGCTGTAGCCAAATTCGTTGT
Human	*CCL2*	CATGAAAGTCTCTGCCGCCC	GGGCATTGATTGCATCTGGCTG
	IL1B	TCCGAGGCACAAGGCACAA	TGGCTGCTTCAGACACTTGAG
	NLRP3	GACCCAGGGATGAGAGTGTTGT	CCCAACCACAATCTCCGAAT
	TNFA	TAGCCCATGTTGTAGCAAACC	ATGAGGTACAGGCCCTCTGAT
	VEGFA	ATAAGTCCTGGAGCGTTCCCT	TTAACTCAAGCTGCCTCGCC
	ICAM1	TGACCGTGAATGTGCTCTCC	TTCCTTTTTGGGCCTGTTGT
	GAPDH	GTTGTCTCCTGCGACTTCA	TGCTGTAGCCGTATTCATTG
	CCL2 ChIP	CCTGGAAATCCACAGGATGC	CGAGAGTGCGAGCTTCAG

Table S3. Descriptive statistics

Figure #	Experimental Groups	Adjusted P Value
Figure 1B	Veh DG (n=4) vs. STZ DMSO (n=4)	0.0002
Figure 1B	STZ DMSO (n=4) vs. STZ DG (n=3)	0.0006
Figure 1D	Veh DG (n=4) vs. STZ DMSO (n=4)	<0.0001
Figure 1D	STZ DMSO (n=4) vs. STZ DG (n=3)	<0.0001
Figure 2A	REDD1+/+ Veh (n=4) vs. REDD1+/+ STZ (n=4)	0.0108
Figure 2A	REDD1+/+ Veh (n=4) vs. REDD1-/- Veh (n=4)	0.0004
Figure 2A	REDD1+/+ STZ (n=4) vs. REDD1-/- STZ (n=4)	<0.0001
Figure 2B	REDD1+/+ Blood glucose vs Urine ACR	<0.0001
Figure 2B	REDD1-/- Blood glucose vs Urine ACR	0.085
Figure 2C	REDD1+/+ Veh (n=5) vs. REDD1+/+ STZ (n=6)	0.0039
Figure 2C	REDD1+/+ STZ (n=6) vs. REDD1-/- STZ (n=5)	0.0012
Figure 2D	REDD1+/+ Veh (n=5) vs. REDD1+/+ STZ (n=5)	<0.0001
Figure 2D	REDD1+/+ STZ (n=5) vs. REDD1-/- STZ (n=5)	<0.0001
Figure 2E	REDD1+/+ Veh (n=4) vs. REDD1+/+ STZ (n=5)	0.0088
Figure 2E	REDD1+/+ STZ (n=5) vs. REDD1-/- STZ (n=5)	0.0066
Figure 2F	REDD1+/+ Veh (n=5) vs. REDD1+/+ STZ (n=6)	<0.0001
Figure 2F	REDD1+/+ STZ (n=6) vs. REDD1-/- STZ (n=5)	<0.0001
Figure 3B	REDD1+/+ Veh (n=3) vs. REDD1+/+ STZ (n=3)	<0.0001
Figure 3B	REDD1+/+ STZ (n=3) vs. REDD1-/- STZ (n=3)	<0.0001
Figure 3D	REDD1+/+ Veh (n=4) vs. REDD1+/+ STZ (n=4)	0.0466
Figure 3D	REDD1+/+ STZ (n=4) vs. REDD1-/- STZ (n=4)	0.0588
Figure 3E	REDD1+/+ Veh (n=4) vs. REDD1+/+ STZ (n=4)	0.0485
Figure 3E	REDD1+/+ STZ (n=4) vs. REDD1-/- STZ (n=4)	0.0428
Figure 3F	REDD1+/+ Veh (n=4) vs. REDD1+/+ STZ (n=4)	0.0025
Figure 3F	REDD1+/+ STZ (n=4) vs. REDD1-/- STZ (n=4)	0.0092

Figure #	Experimental Groups	Adjusted P Value
Figure 4A	REDD1+/+ Veh (n=6) vs. REDD1+/+ STZ (n=6)	<0.0001
Figure 4A	REDD1+/+ STZ (n=6) vs. REDD1-/- STZ (n=6)	<0.0001
Figure 4C	CIHP WT OC (n=6) vs. CIHP WT HG (n=6)	<0.0001
Figure 4C	CIHP WT HG (n=6) vs. CIHP REDD1 KO HG (n=6)	<0.0001
Figure 4E	CIHP WT OC (n=6) vs. CIHP WT HG (n=6)	<0.0001
Figure 4E	CIHP WT HG (n=6) vs. CIHP REDD1 KO HG (n=6)	<0.0001
Figure 4F	IL1B_CIHP WT OC (n=4) vs. CIHP WT HG (n=4)	<0.0001
Figure 4F	IL1B_CIHP WT HG (n=4) vs. CIHP REDD1 KO HG (n=4)	<0.0001
Figure 4F	CCL2_CIHP WT OC (n=4) vs. CIHP WT HG (n=4)	0.0022
Figure 4F	CCL2_CIHP WT HG (n=4) vs. CIHP REDD1 KO HG (n=4)	<0.0001
Figure 4G	CIHP WT OC (n=4) vs. CIHP WT HG (n=4)	<0.0001
Figure 4G	CIHP WT HG (n=4) vs. CIHP REDD1 KO HG (n=4)	<0.0001
Figure 4H	CIHP WT OC (n=3) vs. CIHP WT HG (n=3)	0.0003
Figure 4H	CIHP WT OC (n=3) vs. CIHP REDD1 KO OC (n=3)	0.0002
Figure 4H	CIHP WT HG (n=3) vs. CIHP REDD1 KO HG (n=3)	0.0002
Figure 4I	CIHP WT OC (n=5) vs. CIHP WT HG (n=5)	0.001
Figure 4I	CIHP WT HG (n=5) vs. CIHP REDD1 KO HG (n=5)	0.001
Figure 4J	EV OC (n=4) vs. HA-REDD1 OC (n=4)	0.001
Figure 4J	EV HG (n=4) vs. HA-REDD1 HG (n=4)	<0.0001
Figure 5B	CIHP WT OC (n=3) vs. CIHP WT HG (n=3)	<0.0001
Figure 5B	CIHP WT HG (n=3) vs. CIHP REDD1 KO HG (n=3)	<0.0001
Figure 5D	REDD1 fl/fl Veh (n=9) vs. REDD1 fl/fl STZ (n=9)	0.0043
Figure 5D	REDD1 fl/fl STZ (n=9) vs. REDD1 podKO STZ (n=8)	0.0834
Figure 5F	REDD1 fl/fl Veh (n=6) vs. REDD1 fl/fl STZ (n=6)	0.0001
Figure 5F	REDD1 fl/fl STZ (n=6) vs. REDD1 podKO STZ (n=6)	<0.0001

Figure #	Experimental Groups	Adjusted P Value
Figure 5H	REDD1 fl/fl Veh (n=3) vs. REDD1 fl/fl STZ (n=3)	<0.0001
Figure 5H	REDD1 fl/fl STZ (n=3) vs. REDD1 podKO STZ (n=3)	0.033
Figure 5I	REDD1 fl/fl Veh (n=3) vs. REDD1 fl/fl STZ (n=3)	0.0087
Figure 5I	REDD1 fl/fl STZ (n=3) vs. REDD1 podKO STZ (n=3)	0.01
Figure 6A	CIHP WT OC (n=4) vs. CIHP WT HG (n=4)	<0.0001
Figure 6A	CIHP WT HG (n=4) vs. CIHP REDD1 KO HG (n=4)	0.0001
Figure 6B	CIHP WT OC (n=6) vs. CIHP WT HG (n=6)	<0.0001
Figure 6B	CIHP WT HG (n=6) vs. CIHP REDD1 KO HG (n=6)	<0.0001
Figure 6D	CIHP WT OC (n=6) vs. CIHP WT HG (n=6)	<0.0001
Figure 6D	CIHP WT HG (n=6) vs. CIHP REDD1 KO HG (n=6)	0.0004
Figure 6E	CIHP WT OC (n=12) vs. CIHP WT HG (n=12)	<0.0001
Figure 6E	CIHP WT HG (n=12) vs. CIHP REDD1 KO HG (n=12)	<0.0001
Figure 6F	REDD1 fl/fl Veh (n=5) vs. REDD1 fl/fl STZ (n=5)	<0.0001
Figure 6F	REDD1 fl/fl STZ (n=5) vs. REDD1 podKO STZ (n=5)	<0.0001
Figure 6G	REDD1 fl/fl Veh (n=6) vs. REDD1 fl/fl STZ (n=6)	0.0056
Figure 6G	REDD1 fl/fl STZ (n=6) vs. REDD1 podKO STZ (n=6)	0.0094
Figure 6I	REDD1 fl/fl Veh (n=6) vs. REDD1 fl/fl STZ (n=6)	<0.0001
Figure 6I	REDD1 fl/fl STZ (n=6) vs. REDD1 podKO STZ (n=6)	<0.0001

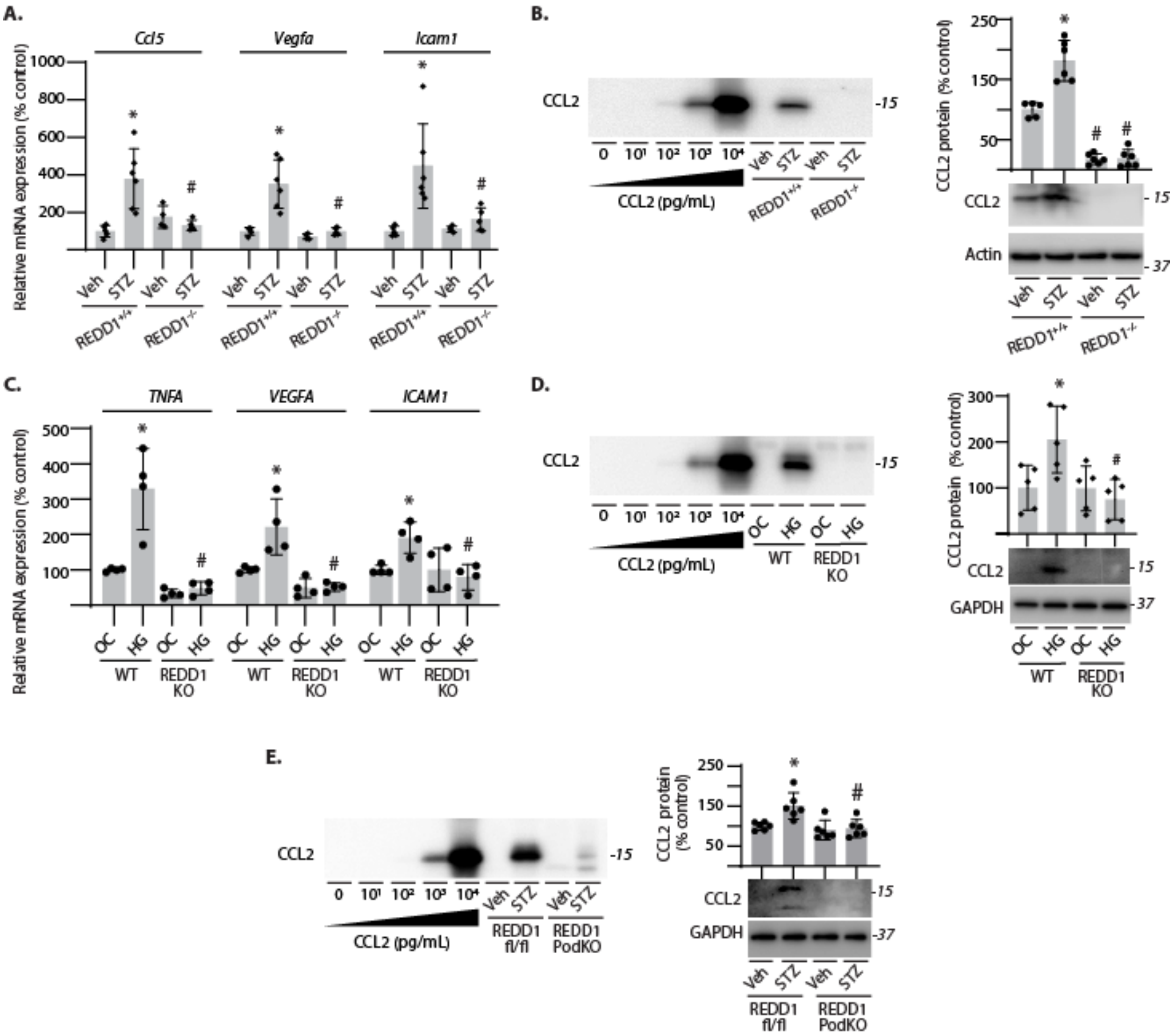

Figure S1. Effect of REDD1 on proinflammatory cytokine expression. *A-B,* Diabetes was induced in REDD1$^{+/+}$ and REDD1$^{-/-}$ mice by streptozotocin (STZ) administration. Non-diabetic control mice received vehicle (Veh). Expression of mRNA transcripts of NF-κB target genes *Ccl5, Vegfa,* and *Icam1* were estimated by qPCR (A). Kidney homogenates were subjected to western blotting and CCL2 protein levels relative to actin were quantified. *C-D,* Wild-type (WT) and REDD1 knockout (KO) CIHP-1 were exposed to culture media containing either 30 mM glucose (HG) or 5 mM glucose plus 25 mM mannitol (OC) for 48 h. mRNA expression of TNFA, VEGFA, and ICAM1 relative to GAPDH were quantified by qPCR (C). CCL2 protein levels were estimated by western blotting (D). E, CCL2 protein levels in kidney homogenates from diabetic (STZ) and nondiabetic (Veh) REDD1$^{fl/fl}$ and REDD1podKO mice were determined by western blotting. Representative blots are shown, and individual data points are plotted. Significance was determined by two-way ANOVA with Tukey's post-hoc analysis. * $p< 0.05$ vs Veh or OC; # $p< 0.05$ vs REDD1$^{+/+}$, WT, or REDD1$^{fl/fl}$.

A.

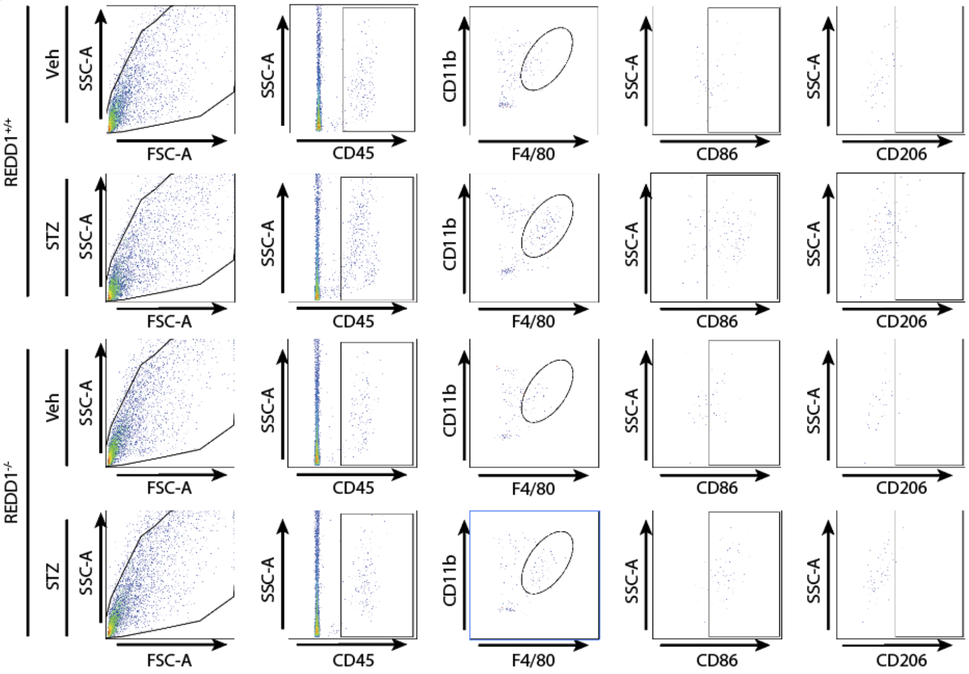

B.

% of total cells				
	CD45+	CD11b+F4/80+	CD86+	CD206+
REDD1$^{+/+}$veh	3.3 ± 0.9	1.2 ± 0.5	0.3 ± 0.2	0.0 ± 0.1
REDD1$^{+/+}$STZ	4.9 ± 1.3 *	2.1 ± 0.4 *	1.4 ± 0.4 *	0.2 ± 0.1
REDD1$^{-/-}$veh	2.5 ± 0.7	0.7 ± 0.1	0.3 ± 0.1	0.0 ± 0.1
REDD1$^{-/-}$STZ	2.6 ± 0.4 #	1.1 ± 0.3 #	0.5 ± 0.1 *#	0.1 ± 0.1

Figure S2. Flow cytometry analysis of REDD1$^{+/+}$ vs. REDD1$^{-/-}$. Diabetes was induced in REDD1$^{+/+}$ and REDD1$^{-/-}$ mice by streptozotocin (STZ) administration. Non-diabetic control mice received vehicle (Veh). *A*, Representative flow cytometry plots show CD11b+F4/80+ macrophage populations within CD45+ leukocytes. The subpopulation of CD86+ and CD206+ cells among CD11b+F4/80+ macrophages are shown. *B*, The population of immune cells as a percent of total cells counted are tabulated. Values are presented as means ± SD (n = 3). Differences between groups were identified by two-way ANOVA. *, $p < 0.05$ versus Veh; #, $p < 0.05$ versus REDD1$^{+/+}$.

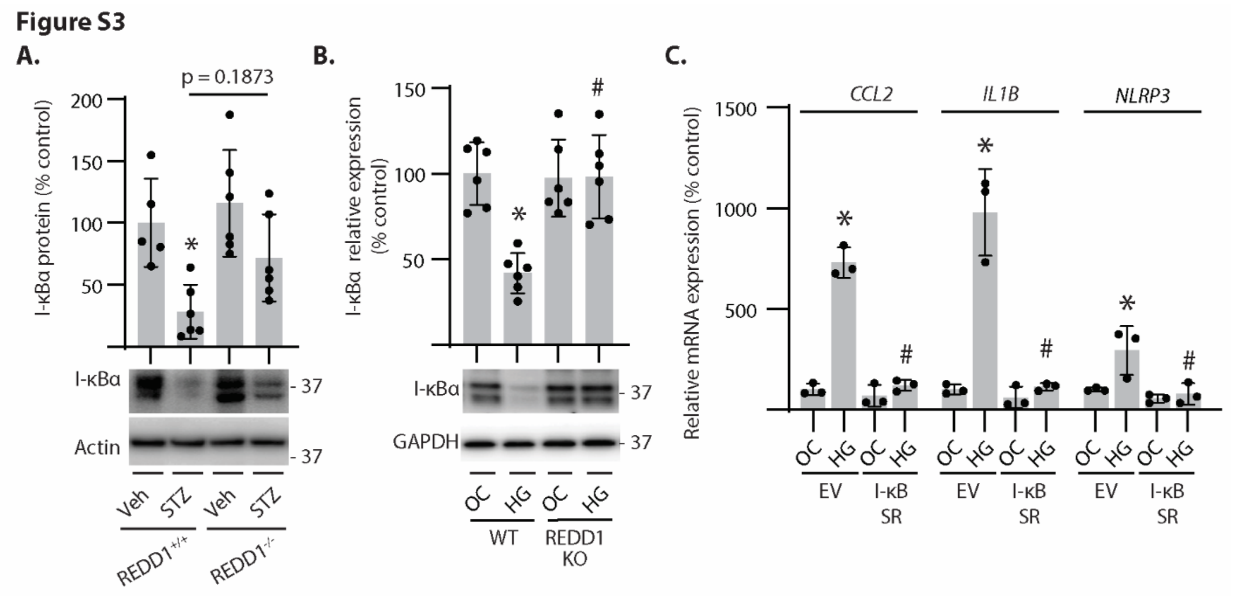

Figure S3. Effect of REDD1 on NF-κB signaling. *A,* Diabetes was induced in REDD1$^{+/+}$ and REDD1$^{-/-}$ mice by streptozotocin (STZ) administration. Non-diabetic control mice received vehicle (Veh). Kidney homogenates were subjected to western blotting and I-κB protein levels relative to actin were quantified. *B-C,* Wild-type (WT) or REDD1 knockout (KO) CIHP-1 were exposed to culture media containing either 30 mM glucose (HG) or 5 mM glucose plus 25 mM mannitol (OC) for 48 h. I-κB protein levels relative to actin were quantified by western blotting (B). *CCL2, IL1B, and NLRP3* mRNA expression was determined in CIHP-1 WT cells expressing either an empty vector (EV) or I-κB super repressor (I-κB SR) plasmid (C). Representative blots are shown, and individual data points are plotted. Significance was determined by two-way ANOVA with Tukey's post-hoc analysis. * p< 0.05 vs Veh or OC; # p< 0.05 vs WT, or EV.

A.

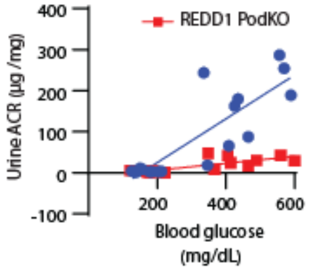

B.

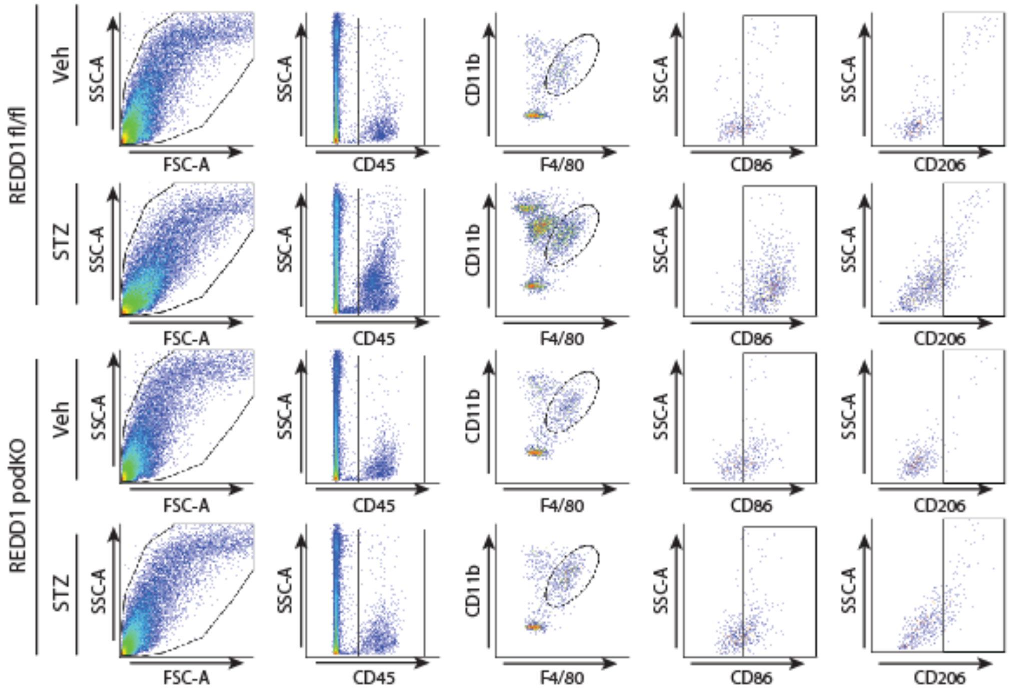

C.

% of total cells				
	CD45+	CD11b+F4/80+	CD86+	CD206+
REDD1$^{fl/fl}$veh	3.3 ± 0.6	1.0 ± 0.3	0.3 ± 0.2	0.2 ± 0.2
REDD1$^{fl/fl}$STZ	7.5 ± 1.6 *	1.9 ± 0.7*	1.5 ± 0.5 *	0.4 ± 0.2
REDD1^{podKO}veh	2.9 ± 0.2	0.9 ± 0.4	0.2 ± 0.1	0.1 ± 0.2
REDD1^{podKO}STZ	3.1 ± 0.2 #	1.5 ± 0.2	0.4 ± 0.1 *#	0.4 ± 0.1

Figure S4. Flow cytometry analysis of REDD1$^{fl/fl}$ vs. REDD1 podKO. REDD1$^{fl/fl}$ and REDD1podKO mice were administered either streptozotocin (STZ) or a vehicle (Veh) control. *A*, Correlation between fasting blood glucose and urine albumin to creatinine ratio (ACR) is shown for REDD1fl/fl mice (blue; Pearson r = 0.93; p<0.0001) and REDD1 PodKO mice (red; Pearson r =0.46; p=0.2519). Representative flow cytometry plots show CD11b+F4/80+ macrophage populations within CD45+ leukocytes. The subpopulation of CD86+ and CD206+ cells among CD11b+F4/80+ macrophages are shown. *B*, The population of immune cells as a percent of total cells counted are tabulated. Values are presented as means ± SD (n = 3). Differences between groups were identified by two-way ANOVA. *, $p < 0.05$ versus Veh; #, $p < 0.05$ versus REDD1$^{fl/fl}$.

Original western blot images-

Figure 1B

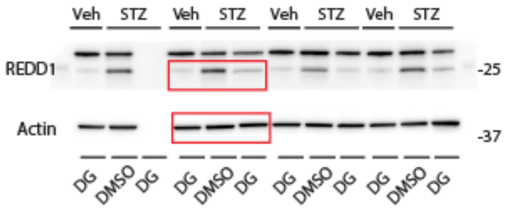

Figure 2B

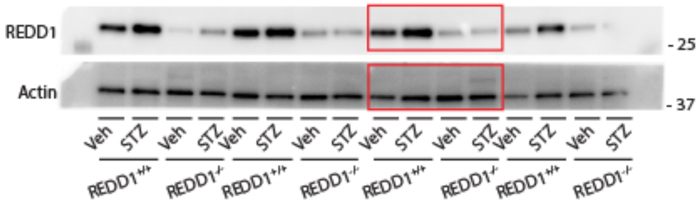

Figure 2H

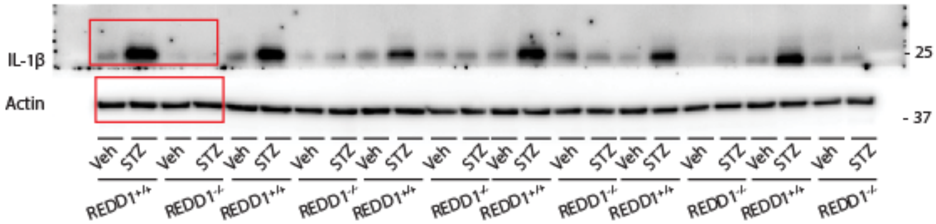

Figure 4A

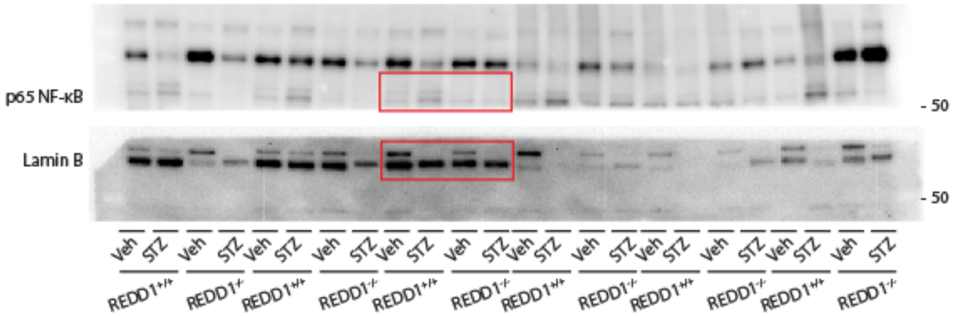

Figure 4B

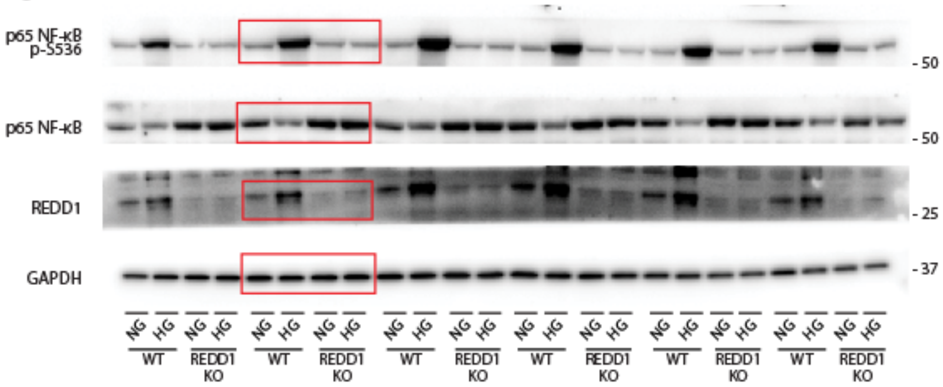

Figure 4J

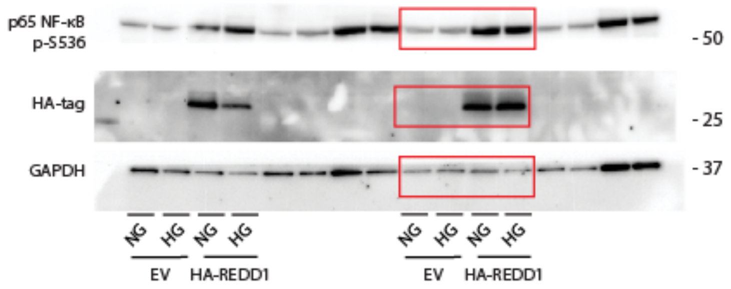

Figure 6B

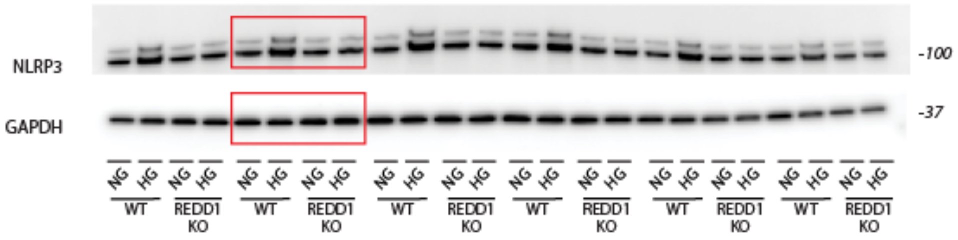

Figure 6D

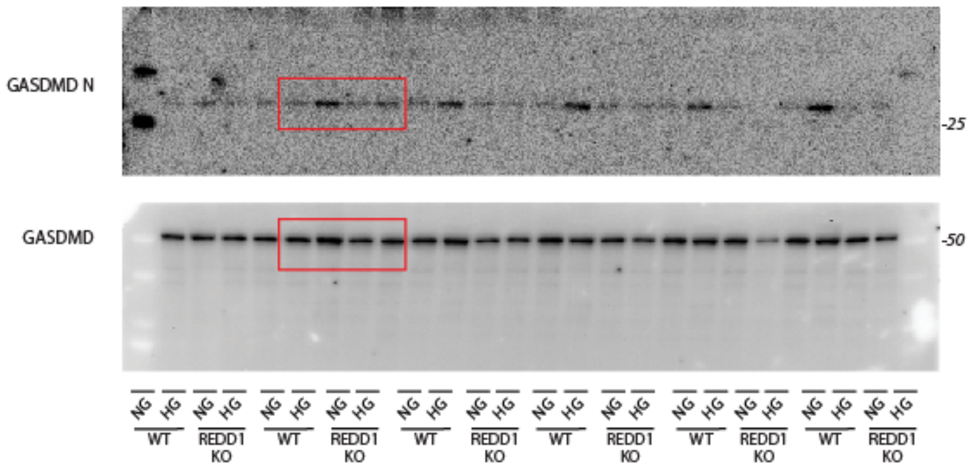

Figure 6G

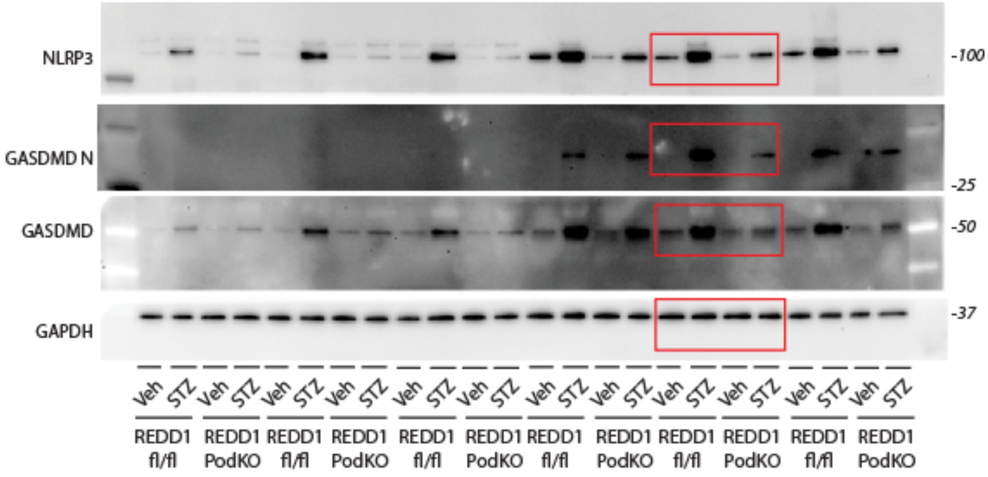

Figure S1B

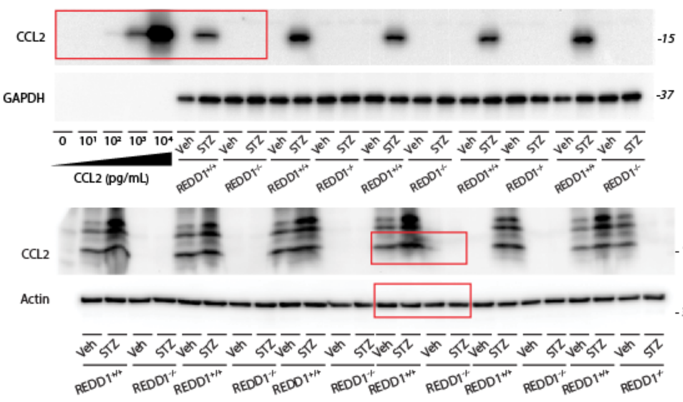

Figure S1D

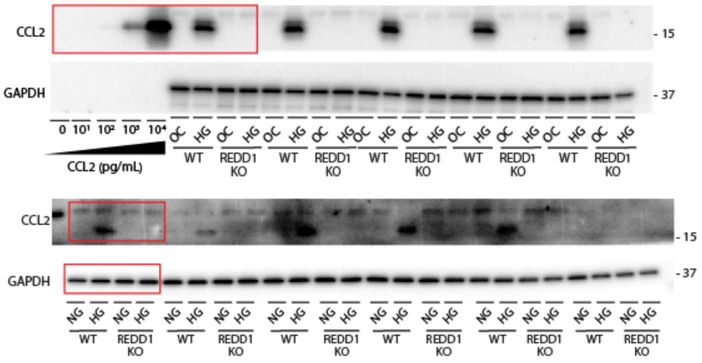

Figure S1E

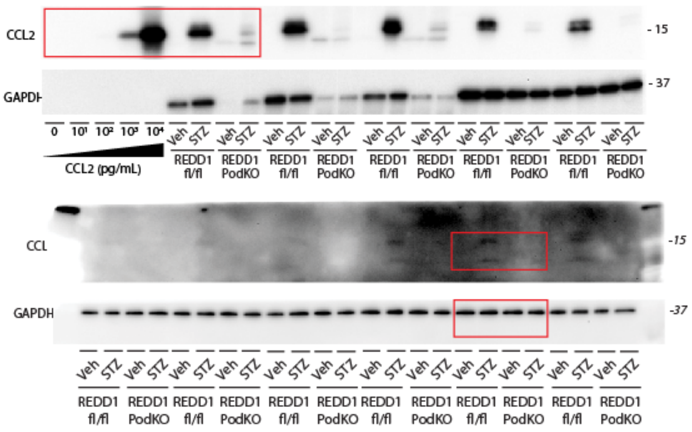

Figure S3A

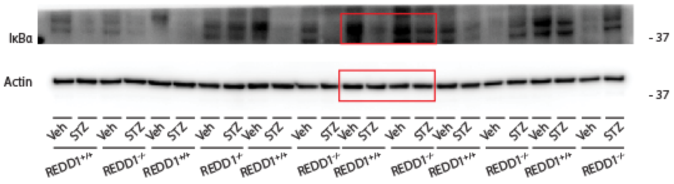

Figure S3B

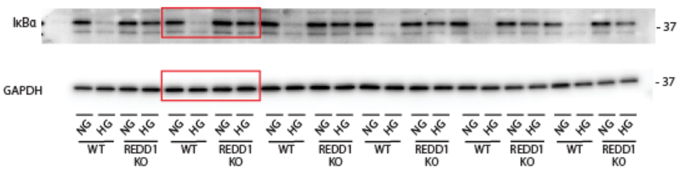

Immunofluorescence individual channels-

Figure 4B

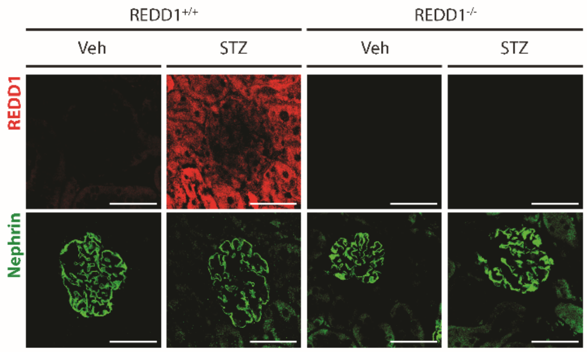

Figure 5D

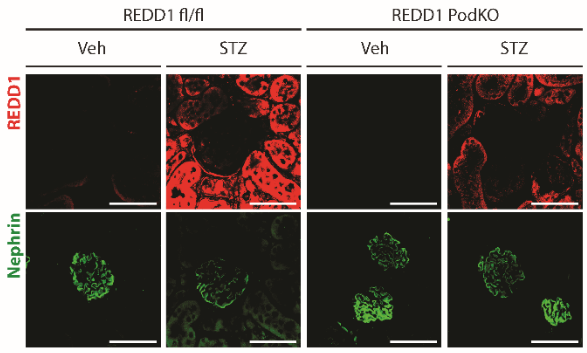

Figure 5F

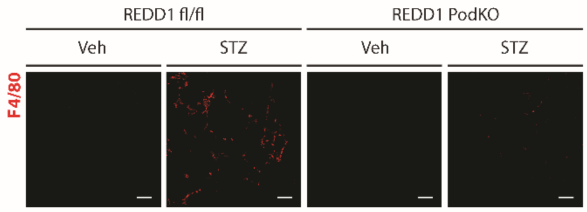

Figure 6C

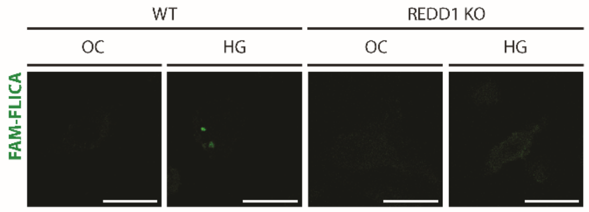

Figure 6H NLRP3

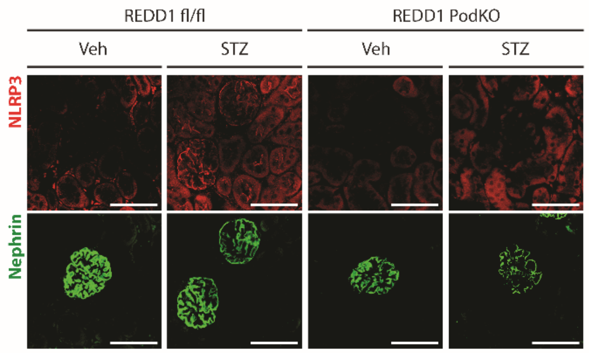

Figure 6H GASDMD

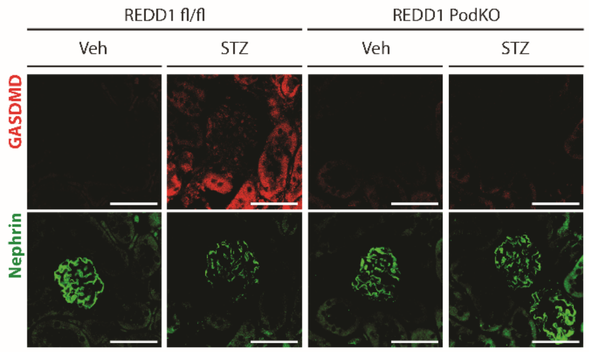

Figure 6H WT-1

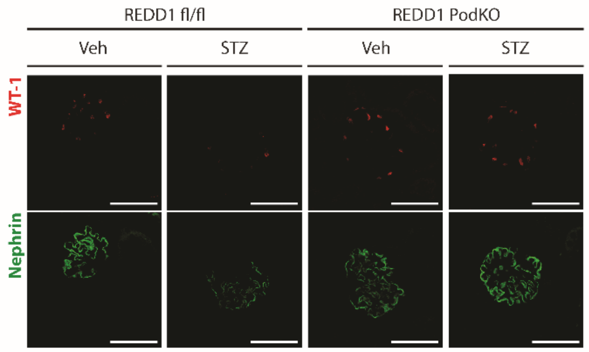

Supplement: Supplementary file 1 — Supplemental Materials [file 41419_2025_7396_MOESM1_ESM.pdf]
